# Identification of an angiogenesis-related risk score model for survival prediction and immunosubtype screening in multiple myeloma

**DOI:** 10.18632/aging.205502

**Published:** 2024-02-05

**Authors:** Manya Yu, Hongquan Ming, Mengting Xia, Jiaqi Fu, Zhiguo Cai, Xing Cui

**Affiliations:** 1 College of Traditional Chinese Medicine, Shandong University of Traditional Chinese Medicine, Jinan, Shandong 250014, China; 2 First Clinical Medical College, Shandong University of Traditional Chinese Medicine, Jinan, Shandong 250014, China; 3 Department of Quality Control, Affiliated Hospital of Shandong University of Traditional Chinese Medicine, Jinan, Shandong 250014, China; 4 Department of Oncology and Hematology, The Second Affiliated Hospital of Shandong University of Traditional Chinese Medicine, Jinan, Shandong 250001, China

**Keywords:** multiple myeloma, angiogenesis, immune microenvironment, prognosis, immunotherapy

## Abstract

Background: Multiple myeloma (MM) is an incurable B-cell malignancy, but with the emergence of immunotherapy, a potential cure is hopeful. The individualized interaction between the tumor and bone marrow (BM) microenvironment determines the response to immunotherapy. Angiogenesis is a constant hallmark of the BM microenvironment in MM. However, little is known about the potency ability of angiogenesis-associated genes (AAGs) to regulate the immune microenvironment of MM patients.

Methods: We comprehensively dissected the associations between angiogenesis and genomic landscapes, prognosis, and the immune microenvironment by integrating 36 AAGs. Immunohistochemistry was performed to verify the correlation between angiogenic factor expression and patient prognosis. Single-sample gene set enrichment analysis was applied to quantify the relative abundance of 28 infiltrating cells. The AAG score was constructed using the least absolute shrinkage and selection operator Cox regression model.

Results: Angiogenesis was closely correlated with MM patient prognosis, and the mutation intensity of the AAGs was low. Immunohistochemistry confirmed that high microvessel density predicted poor prognosis. Three AAG clusters and two gene clusters with distinct clinical outcomes and immune characteristics were identified. The established AAG_score model performed well in predicting patient prognosis and active immunotherapy response. The high-AAG_score subgroup was characterized by reduced immune cell infiltration, poor prognosis, and inactive immunotherapy response. Multivariate analyses indicated that the AAG_score was strongly robust and independent among the prognostic variables.

Conclusion: This study revealed that angiogenesis is significantly related to MM patient prognosis and immune phenotype. Evaluating the AAG signature was conducive to predicting patient response to immunotherapy and guiding more efficacious immunotherapy strategies.

## INTRODUCTION

Multiple myeloma (MM) is a B-cell malignancy characterized by the proliferation of clonal plasma cells in the bone marrow (BM) and is largely incurable [1]. Over the past 20 years, the incidence of MM has increased by 126% worldwide [2]. The increasing prevalence and high recurrence rate of MM make it a major and urgent health challenge. Neovascularization in the BM fuels the progression of MM, and a marked correlation was observed between the BM microvascular density and progression-free survival (PFS) and overall survival (OS) in MM patients [3]. Additionally, a notable decrease in BM microvascular density is evident in patients who successfully achieve remission, in contrast to patients who exhibit no response to therapy [4]. Although antiangiogenic drugs have demonstrated significant efficacy in animal models, their efficacy in humans has been less satisfactory. These treatments lead to a moderate extension of PFS followed by tumor relapse [5]. Additional research is crucial to refine treatment regimens and deepen our understanding of tumor angiogenesis and the mechanisms driving resistance development.

The efficacy limitations and treatment resistance of antiangiogenic therapies may be mediated by increased hypoxia and acidosis, potentially due to excessive pruning of tumor vessels by anti-VEGF therapy in a time- and dose-dependent manner [6, 7]. In addition to affecting cancer cells and the delivery and efficacy of anticancer drugs, hypoxia and acidosis also severely impair the function of immune effector cells, such as by compromising APC functionality and stimulating T-cell responses [8]. Furthermore, hypoxia increases the expression of SDF1-α and CCL28, inducing an immunosuppressive tumor microenvironment by recruiting Tregs, MDSCs, and M2-type TAMs, consequently initiating a tolerant state [9–12]. Given the recent success of immunotherapies, combinations of antiangiogenic agents with immunotherapies have become an attractive strategy [13–15]. However, implementing such combinations requires a better understanding of their interactions and exploring methods for selecting patients likely to respond to this therapy. Therefore, a comprehensive analysis of the relationship between angiogenesis and the immune microenvironment is crucial. Because the majority of studies have focused on one or two angiogenic genes and individual immune cells, our understanding of the overall infiltration characteristics of the BM immune microenvironment mediated by multiple angiogenic molecules in MM is limited.

In the present study, we systematically investigated the expression of angiogenesis-associated genes (AAGs) and their effects on the clinical features, immune landscape, and therapeutic response of MM patients. We identified three distinct AAG subtypes of MM via the Gene Expression Omnibus (GEO) database. Different immune characteristics and biological functions were observed among these subgroups. Furthermore, for the first time, we propose an AAG_score model for MM that integrates the AAG subtype and immune features. The model has great robustness and independence in helping us predict the clinical prognosis and immunotherapeutic response of MM patients.

## MATERIALS AND METHODS

### Data preprocessing

Gene expression profiles, somatic mutation data, and associated clinicopathological data of MM patients were retrieved from the Multiple Myeloma Research Foundation (MMRF) program (https://research.themmrf.org and http://www.themmrf.org). GSE24080 and GSE5900 from the GEO repository were utilized to acquire clinical parameters and normalized gene expression data [16, 17]. Patients lacking significant clinicopathological or survival information were excluded from further analyses. Thirty-six AAGs were obtained from the MSigDB Team (Hallmark Gene set) (Supplementary Table 1). The maftools R package was used to analyze and visualize the masked somatic mutation data [18].

### Identification of expression patterns of AAGs

According to the expression of 35 AAGs (for which VTN was excluded because it was undetected), an unsupervised clustering analysis was carried out to identify distinct angiogenesis-related patterns. The number and robustness of the clusters were assessed by a consensus clustering algorithm [19]. The R package ConsensusClusterPlus executed the above steps in 1000 iterations to ensure the robustness of the classification [20]. The gene set variation analysis (GSVA) algorithm was applied to the Kyoto Encyclopedia of Genes and Genomes (KEGG) gene set (c2.cp.kegg.v7.4) to determine the biological functional differences in the AAG-related clusters [21].

### Immunohistochemistry (IHC)

After written informed consent was obtained, IHC targeting of human CD34 and VEGFA proteins was performed on 4-μm-thick bone marrow specimens from 20 MM patients hospitalized at the Affiliated Hospital of Shandong University of Traditional Chinese Medicine from July 2020 to July 2022 following informed and consent-approved protocols from the ethics committee of the Affiliated Hospital of Shandong University of Traditional Chinese Medicine (2020) (ethical review No. (010) - KY). After dewaxing and rehydration, the sections were incubated with 0.3% H2O2 formaldehyde to inhibit endogenous peroxidase activity and subjected to microwave antigen retrieval and cooled at room temperature. After the cells were incubated with 10% goat serum, primary antibodies, including mouse anti-human CD34 (1:200; Servicebio, China) and mouse anti-human VEGFA (1:200; Servicebio, China), were applied. After washing, the sections were incubated with a biotinylated anti-mouse secondary antibody and labeled with streptavidin-peroxidase solution (Servicebio). The presence of antigen was visualized by staining sections with DAB (Servicebio) and counterstaining sections with hematoxylin (Servicebio).

### Evaluation of VEGFA expression and microvessel density (MVD)

The determination of the plasma cell percentage in VEGFA-stained bone marrow sections was performed manually. The calculation formula for the H score was as follows: H score = intensity of staining × % positivity. Megakaryocytes strongly express VEGF, so they served as internal positive controls [22].

The degree of angiogenesis was assessed using CD34-labeled microvessels. The regions with the highest number of microvessels were identified at 100× magnification and then converted to 400× magnification to measure the microvessel numbers in each region. The average was obtained as the MVD for each region [23].

### Associations between molecular patterns and the clinical characteristics and prognosis of MM patients

To determine the clinical significance of the clusters by consensus clustering, we investigated the associations between molecular patterns and clinical features and between molecular patterns and survival outcomes. The clinical variables included age, gender, type, MRI, LDH, ALB, and HGB. Moreover, the differences in event-free survival (EFS) and overall survival (OS) between patients with different patterns were evaluated via Kaplan–Meier (K–M) analyses via the survival and survminer R packages [24].

### Correlations between molecular patterns and immune characteristics

We used single-sample gene set enrichment analysis (ssGSEA) to assess the immune microenvironment based on the immune gene sets (Supplementary Table 2) obtained from the research of Charoentong [25], which can provide a better picture of tumor conditions than CIBERSORT when tumor cells are present in similar proportions. The expression of immune checkpoints (ICPs), including PD-L1, CTLA4, PD-1, PD-L2, LAG3, and HAVCR2, was subsequently compared between the different groups. Additionally, the correlations between the AAG_score and the differentially expressed ICPs were calculated.

For MM, most related studies have focused on CD138-positive cells obtained via magnetic cell sorting (MACS). Due to the limitations of the sorting methods, the remaining nontumor cells were randomly mixed in the extracted samples, still characterizing the matrix environment of MM in a relatively large sample quantity [26].

### Identification of immune-related differentially expressed genes (DEGs) and functional annotation

The limma R package was used to determine the DEGs between distinct angiogenesis clusters with the cutoff criteria |logFC| ≥ 1 and *p* value < 0.05 [27]. To explore the potential biological processes related to the DEGs, Gene Ontology (GO) and Kyoto Encyclopedia of Genes and Genomes (KEGG) analyses were performed using the clusterProfiler R package [28]. The immune-related DEGs were determined by the intersection of the immune gene set (Supplementary Table 2) and the DEGs.

### Generation of the angiogenesis-associated prognostic AAG_score and nomogram

We performed K–M analysis for immune-related DEGs, and 26 genes associated with both OS and EFS were selected with the criterion of *p* < 0.01. Based on the combined role of these 26 genes in MM progression, the AAG_Score signature was constructed to comprehensively assess the role of these molecules in patient prognosis, the immune microenvironment, and immunotherapy response. Least absolute shrinkage and selection operator (LASSO) Cox regression was used to reduce the dimensionality, and the 11 most stable molecules were selected to construct the AAG_score signature with the glmnet R package [29]. The formula is as follows:

AAG_score = (0.2874 × expression of IFI16) + (−0.1035 × expression of STAP1) + (0.1546 × expression of GEMIN6) + (−0.2298 × expression of SLC7A7) + (−0.0694 × expression of LST1) + (−0.0011 × expression of IGHM) + (−0.0710 × expression of FUCA1) + (−0.0009 × expression of PD-L2) + (0.1405 × expression of NUF2) + (−0.1112 × expression of CD22) + (−0.0192 × expression of ADAM28).

The AAG_score model was validated in the test set and the entire cohort. Cox regression analyses were subsequently performed to determine the independent prognostic factors. The risk prediction model was subsequently constructed as a nomogram according to the independent prognostic factors. Calibration curve analysis and decision curve analysis (DCA) were performed to evaluate the performance of the constructed nomogram.

### Acquisition of immunotherapeutic cohorts

The IMvigor210 immunotherapeutic cohort with RNA-seq data and complete clinical data was included in our study [30]. The IMvigor210 study investigated the efficacy of an anti-PD-L1 antibody in advanced or metastatic urothelial carcinoma patients. The complete transcriptome profile and clinical information were downloaded from http://research-pub.gene.com/IMvigor210CoreBiologies/, and the count was converted to transcripts per kilobase million (TPM) values. The DEseq2 R package was used for normalization.

### Statistical analysis

Comparisons between two groups were performed using the Wilcoxon test. One-way ANOVA and the Kruskal–Wallis test were used to analyze differences among three or more groups. Spearman and distance correlation analyses were used for correlation analyses. All the statistical analyses were performed using R software (version 4.1.0) and its relevant packages. *P* < 0.05 was regarded as statistically significant.

## RESULTS

### Differential expression and genetic mutation landscape of AAGs in MM

**Figure 1:**
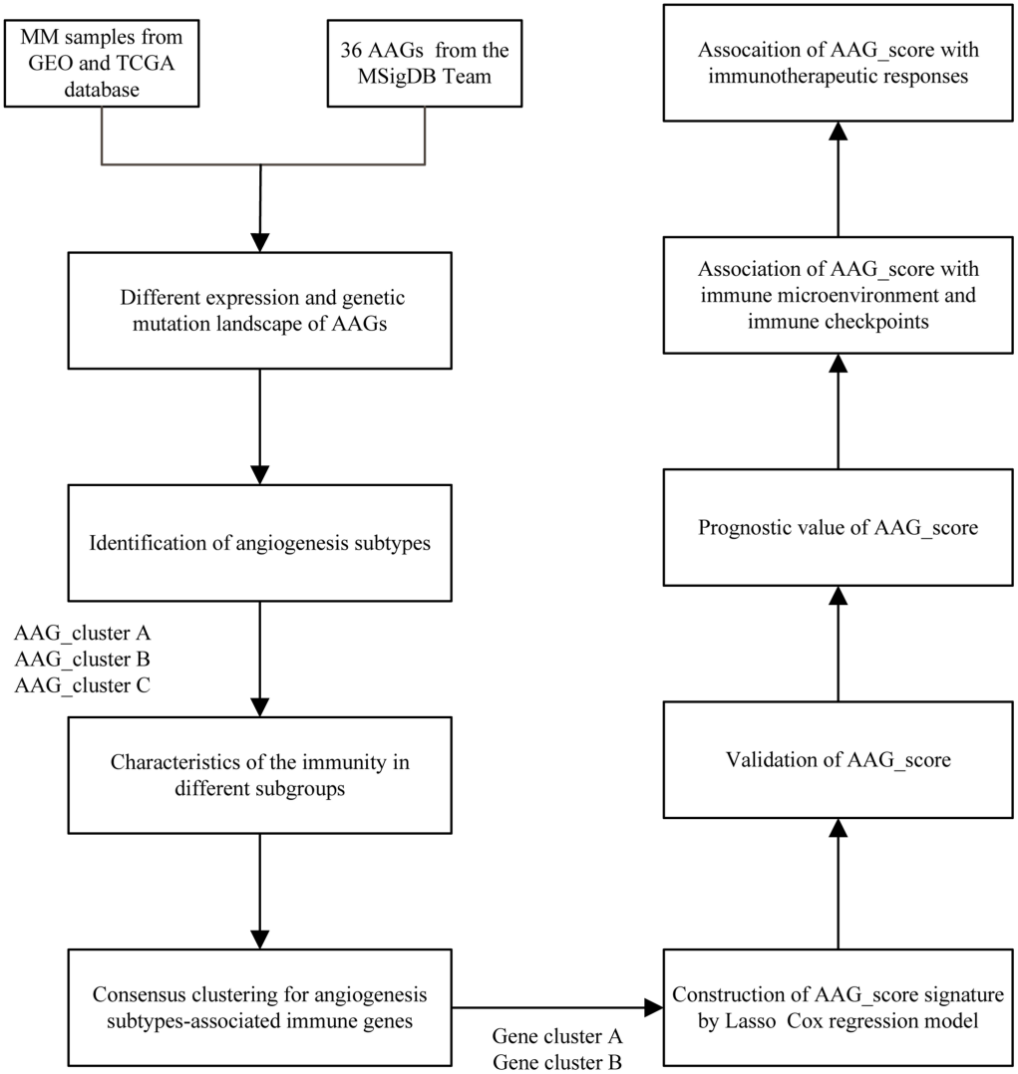
The entire analytical process of the study.

**Figure 2:**
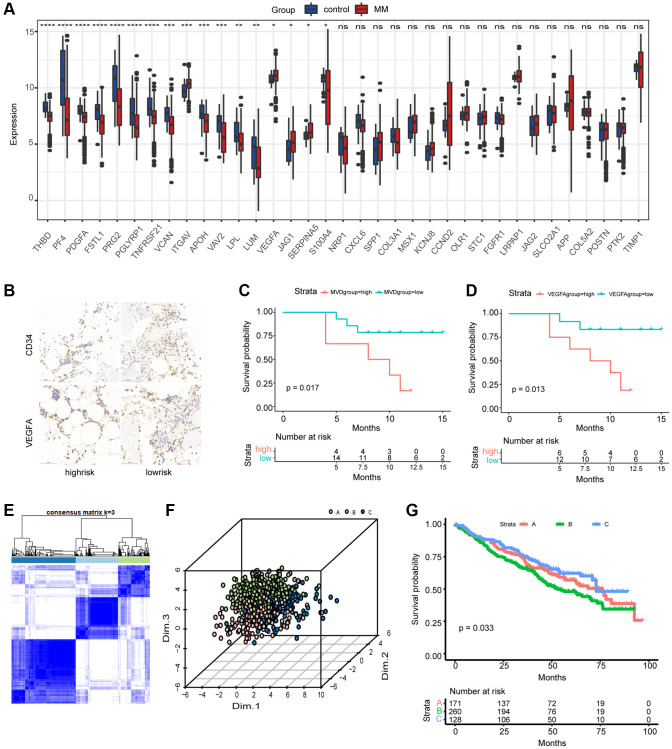
**Differential expression of AAGs in MM and generation of angiogenesis subgroups.** (**A**) Differences in the expression of AAGs between MM patients and healthy controls (^*^P < 0.05; ^**^P < 0.01; ^***^P < 0.001; ^****^P < 0.0001). (**B**) Representative images of MM patient BM tissues stained with CD34 and VEGFA antibodies (scale bar, 50 μm). Overall survival analyses of the MVD group (**C**) and VEGFA group (**D**) using Kaplan–Meier curves. (**E**) Consensus matrix heatmap depicting three gene clusters associated with the AAGs. The colors indicate the similarity or dissimilarity among samples at each iteration, with blue indicating higher similarity and white indicating lower similarity. (**F**) PCA based on the AAGs revealed three disjointed populations. Red, cluster A samples; green, cluster B samples; blue, cluster C samples. (**G**) OS analysis of patients in the AAG_clusters using Kaplan–Meier curves.

The detailed flowchart of this work is shown in Figure 1. We first evaluated the expression levels of the 35 AAGs in MM samples and normal samples in the GSE24080 and GSE5900 datasets. A total of 17 DEGs were found (Figure 2A). A protein–protein interaction (PPI) network was constructed through Mode to reveal the hub genes (Supplementary Figure 1A); among these genes, only VEGFA and ITGAV were significantly upregulated in MM patients. Immunohistochemistry was used to examine the relationship between MVD and the expression of VEGFA, a key molecule involved in angiogenesis, and survival (Figure 2B). The Surv_cutpoint function was used to determine the optimal cutoff points for VEGFA (0.7993967) and MVD (1.5243902) in the datasets, dividing the patients into high- and low-expression groups. K–M analyses revealed that patients in the groups with a lower MVD and lower expression of VEGFA had better survival (Figure 2C, 2D).

Next, we selected bone marrow samples from primary MM patients from the MMRF to evaluate the degree of variation. The findings revealed that missense mutations, SNPs, and C>T mutations were more common, with the highest mutation frequency being 1883 (Supplementary Figure 1B). Furthermore, we explored somatic mutations in AAGs in MM. As the waterfall diagram depicted (Supplementary Figure 1C), only 78 of the 724 (10.77%) MM samples presented genetic mutations, and the findings suggested that VCAN and COL3A1 are the genes with the highest mutation rates among the AAGs. These findings indicated the potential role of AAGs, especially the hub genes VEGFA and ITGAV, in MM pathogenesis, despite the low mutation intensity of these AAGs.

### Generation of angiogenesis subgroups in MM

A total of 559 MM patients from the GSE24080 dataset were enrolled in this study to determine the relationship between angiogenesis and clinical characteristics. To explore the relationship between the expression patterns of AAGs and MM subtypes, we performed a consensus clustering analysis to classify MM patients according to the expression levels of these AAGs. The optimal number of clusters was determined by evaluating the quality of clustering based on consensus values, the cumulative distribution function, and the proportion of ambiguous clustering. Our findings indicated that the optimal clustering variable was 3 (Figure 2E), and MM patients in the entire cohort were well dispersed in cluster A (*n* = 171), cluster B (*n* = 260), and cluster C (*n* = 128). Principal component analysis (PCA) also revealed three distinct populations (Figure 2F). The clinicopathological variables of these three clusters are shown in Supplementary Figure 2A. Furthermore, the EFS and OS times of patients in the AAG clusters were explored, and a significant difference in EFS was observed (Figure 2G); however, the results revealed no significant difference in OS. Additionally, GSVA was performed, as displayed in Supplementary Figure 2B; cluster C was enriched in hematopoiesis-associated pathways, metastasis-associated pathways, and immune-associated pathways, such as hematopoietic cell lineage, cell adhesion molecules (CAMs), ECM receptor interaction, cytokine receptor interaction, complement and coagulation cascade, allograft rejection, and the intestinal immune network. Therefore, these three AAG clusters exhibited substantial differences in clinical characteristics and biological functions.

### Characteristics of immunity in different subgroups

**Figure 3:**
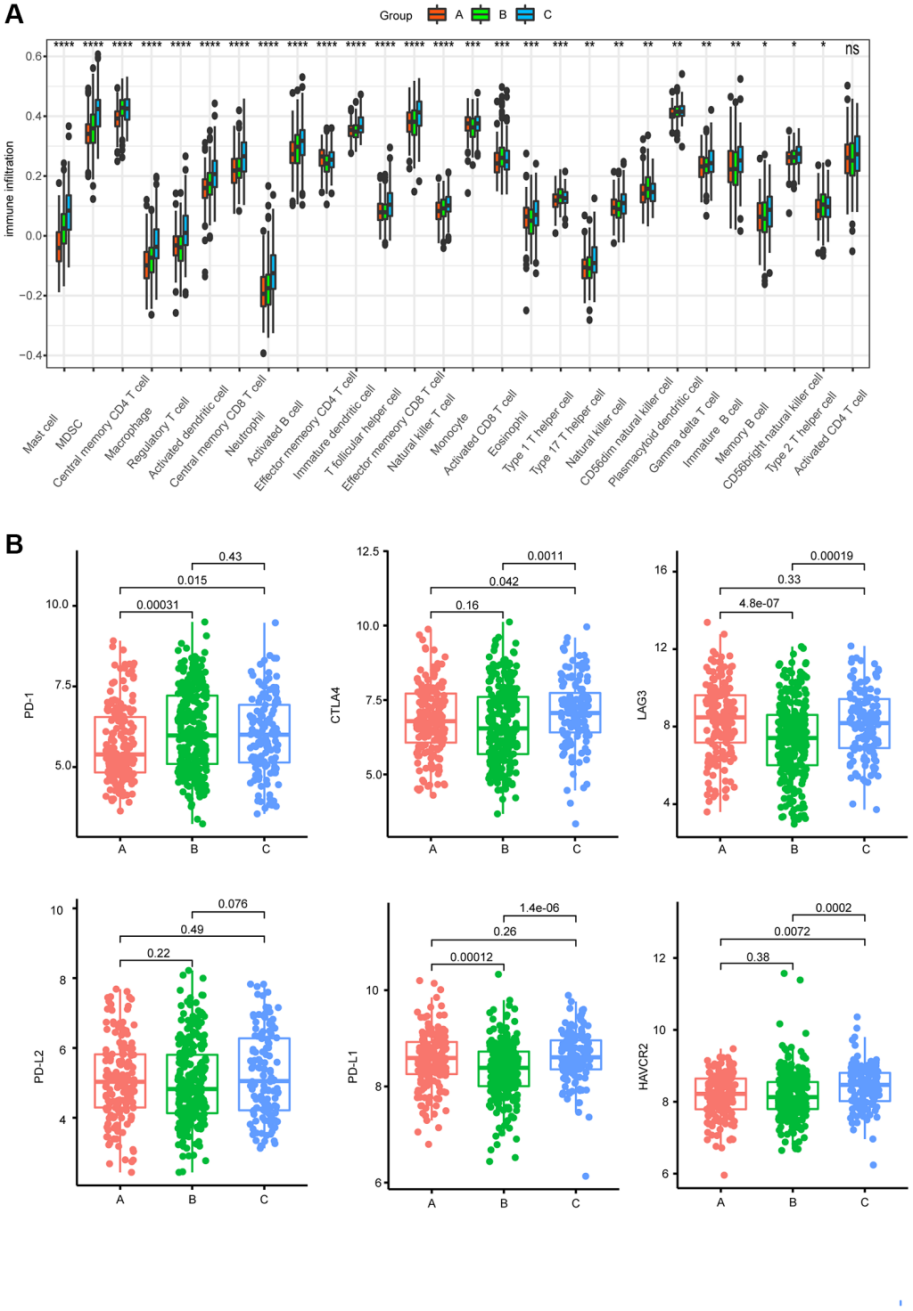
**Characteristics of immunity in different subgroups.** (**A**) Differences in 28 infiltrating immune cells among the three AAG clusters (^*^P < 0.05; ^**^P < 0.01; ^***^P < 0.001; ^****^P < 0.0001). (**B**) Expression of 6 ICPs in the three subtypes.

To determine the relationship between AAGs and the immune characteristics of MM patients, we analyzed the associations between the three clusters and 28 immune metagenes. As shown in Figure 3A, a significant difference was found in the enrichment of the 28 immune metagenes among the three clusters, most of which exhibited high expression in cluster C cells, especially mast cells, myeloid-derived suppressor cells (MDSCs), macrophages, regulatory T cells, activated dendritic cells, central memory CD8 T cells, neutrophils, activated B cells, immature dendritic cells, T follicular helper cells, effector memory CD8 T cells, natural killer T cells, and eosinophils. Furthermore, we investigated the associations between the three clusters and the expression of the 6 ICPs. The expression levels of CTLA4 and HAVCR2 in cluster C were significantly greater than those in the other clusters, while cluster B presented lower expression levels of PD-L1 and LAG3 (Figure 3B).

### Identification of gene subgroups based on DEGs

**Figure 4:**
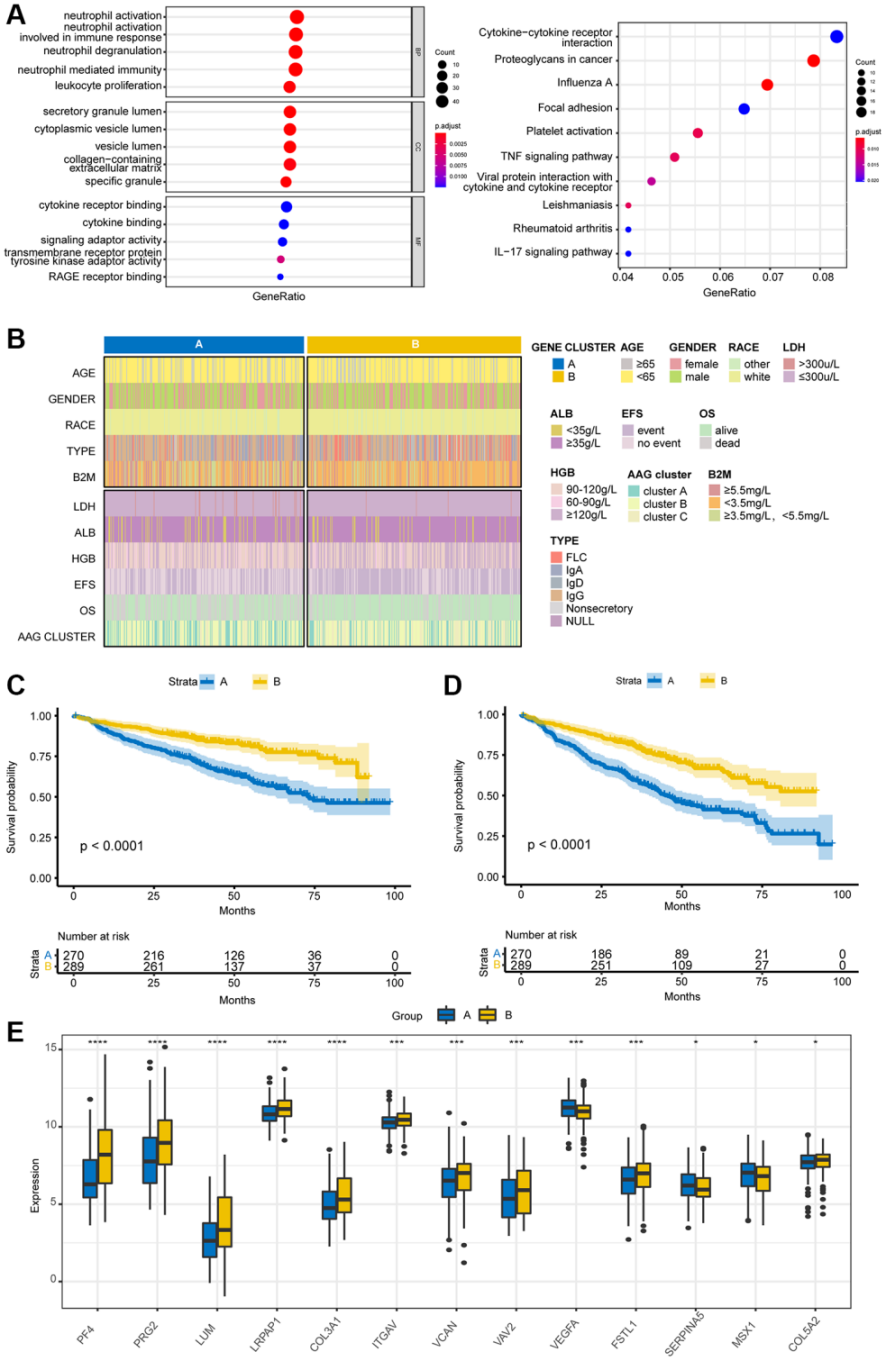
**Identification of gene subgroups based on DEGs.** (**A**) GO and KEGG pathway enrichment analyses of DEGs among the three AAG clusters. (**B**) Cluster diagram of clinical characteristics between the two gene clusters. Overall survival (**C**) and event-free survival (**D**) analyses of patients stratified according to gene cluster analysis using Kaplan–Meier curves. (**E**) Differences in the expression of AAGs between the two gene clusters (^*^P < 0.05; ^**^P < 0.01; ^***^P < 0.001).

To investigate the biological reactions in the three angiogenesis subgroups, we obtained DEGs among the three AAG clusters with the “limma” package and conducted GO and KEGG analyses. GO enrichment analysis demonstrated that these genes were enriched mainly in immune-associated biological processes (Figure 4A). KEGG analysis revealed the enrichment of immune- and cancer-associated pathways, indicating that angiogenesis is a key factor in regulating immunity and tumorigenesis. Then, we separated the immune-related genes from the DEGs and utilized K–M analyses to screen genes associated with survival. Finally, 26 genes associated with both OS and EFS were selected with the criterion of *p* < 0.01. To study the specific regulatory mechanisms involved, the patients were divided into two different gene clusters according to 26 prognostic genes by a consensus clustering method. The clinical characteristics of the two groups are shown in Figure 4B. K–M analyses revealed that patients with the A subtype had shorter OS and EFS than patients with the B subtype did (Figure 4C, 4D). Additionally, a boxplot of angiogenesis-related genes is presented in Figure 4E; only VEGFA, SERPINA5, and MSX1 were expressed at higher levels in the A subtype than in the B subtype.

### Construction and validation of the prognostic AAG_score model

**Figure 5:**
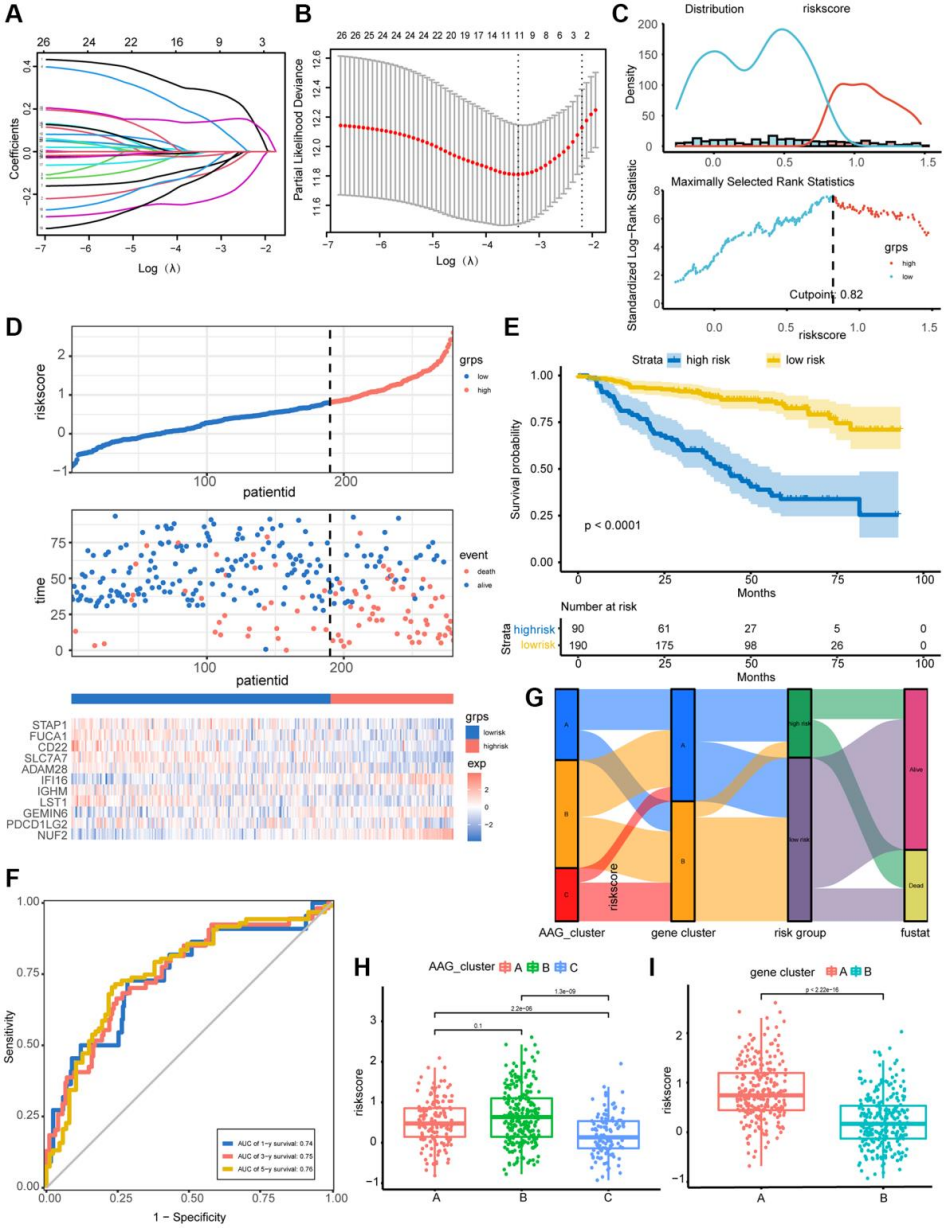
**Construction and validation of the prognostic AAG_score model.** (**A**) LASSO coefficient profiles of 26 DEGs related to immunity and prognosis. (**B**) Tenfold cross-validation for tuning parameter selection in the LASSO model. According to log(λ), the partial likelihood deviation graph was drawn, where λ is the tuning parameter. Partial likelihood deviation values are displayed, and the error bars indicate SEs. Dotted vertical lines are drawn at the optimal values according to the minimum criteria and 1-SE criteria. (**C**) The optimal cutoff point for dichotomizing patients into low and high AAG_score groups were determined by the survminer R package. The optimal cutoff value was 0.82. (**D**) Percentage of deaths in the high- and low-risk groups as the AAG_score increased. Expression patterns of 11 selected molecules in different risk groups. (**E**) Overall survival analysis of risk groups using Kaplan–Meier curves. (**F**) ROC analysis of the ability of the AAG_score to predict 1-, 3-, and 5-year OS and its specificity. (**G**) Alluvial diagram showing the changes in the AAG clusters, gene clusters, AAG_scores, and clinical outcomes. (**H**) Differences in the AAG_scores among the three AAG clusters. (**I**) Differences in the AAG_scores between the two gene subgroups.

A prognostic model combining angiogenesis and immune features was developed based on the 26 immune-related DEGs. A total of 559 MM patients were randomly assigned to a training set (*n* = 275) or a test set (*n* = 274). The LASSO Cox regression model was used to screen the 11 most representative immune-related molecules concerning patient prognosis (Figure 5A, 5B). The results of univariate Cox analysis of the selected molecules are shown in Supplementary Table 3. With a cutoff value of 0.82 determined by the survminer R package (Figure 5C), patients in the training set were classified into high- and low-risk groups. As the AAG_score increased, patient mortality increased notably. A heatmap was constructed to show the expression of 11 representative genes in the two AAG_score groups (Figure 5D). Additionally, K–M analysis indicated that low-risk patients had a survival advantage over high-risk patients (Figure 5E), and the areas under the receiver operating characteristic (ROC) curves (AUCs) of 1-, 3-, and 5-year OS were 0.74, 0.75, and 0.76, respectively, indicating the perfect performance of the AAG_score in the training set (Figure 5F).

We then investigated the distribution of all patients in three AAG clusters, two gene clusters, and two AAG score groups (Figure 5G). The AAG_score was highest in AAG_cluster B and lowest in AAG_cluster C (Figure 5H). The performance of the gene clusters was similar to that of the AAG clusters (Figure 5I), indicating that the higher the AAG_score was, the worse the survival was. Based on the above results, we concluded that the AAG_score was a good predictor of prognosis in MM patients.

The AAG_score was further tested in the test set and the entire set depending on the cutoff of the training set, and similar results were obtained. K–M survival analyses revealed that, compared with patients in the high-risk group, patients in the low-risk group had superior survival. The AUC still indicated good performance (Supplementary Figures 3 and 4).

### Correlations between the AAG_score and clinical features

**Figure 6:**
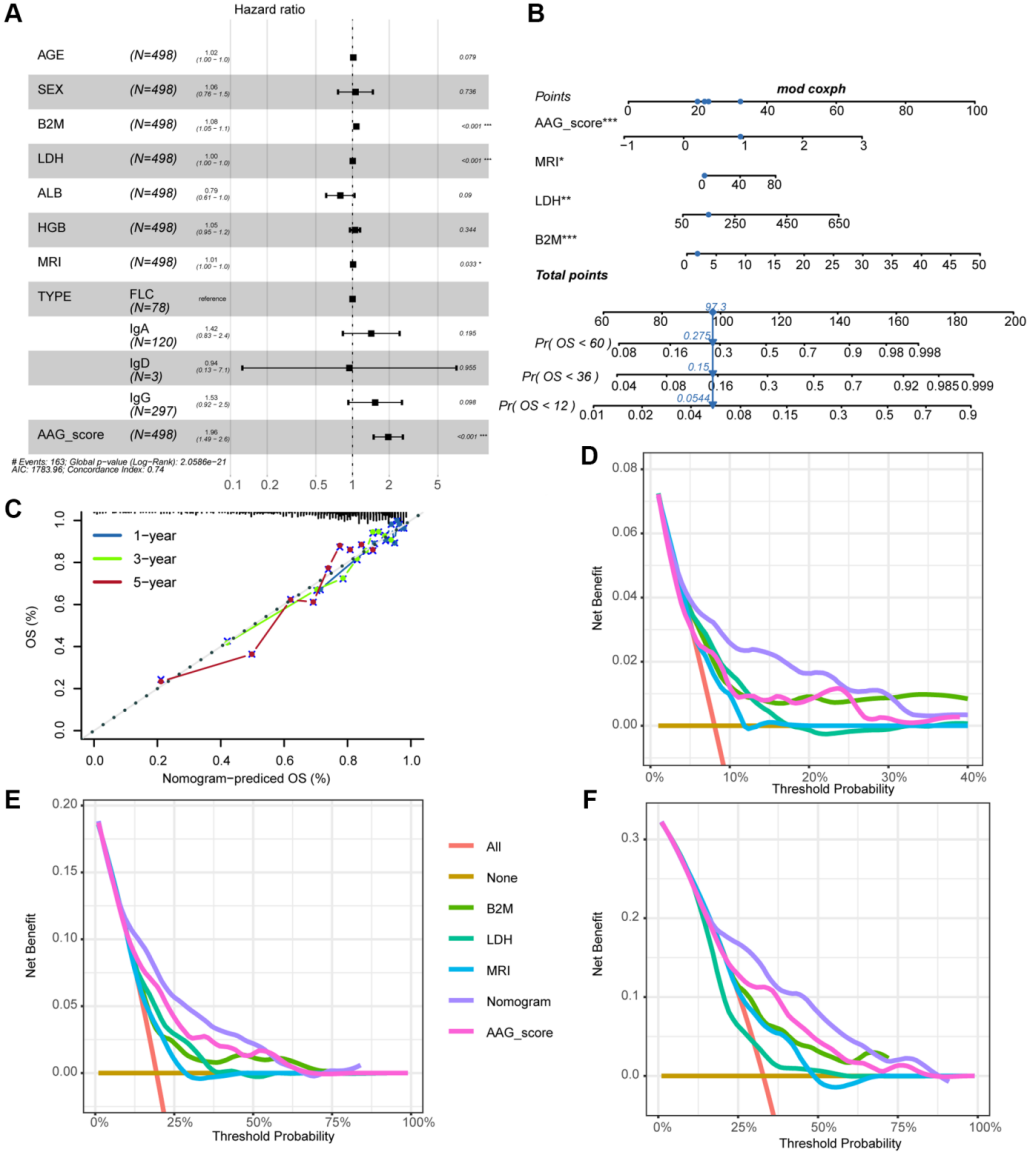
**Correlations between the AAG_score and clinical features.** (**A**) Forest plot showing that the AAG_score was an independent prognostic biomarker according to multivariate analyses of the entire cohort. (**B**) Nomogram for predicting the 1-, 3-, and 5-year OS of MM patients. (**C**) The 1-, 3-, and 5-year calibration plots of the nomogram. (**D**–**F**) The 1-, 3-, and 5-year decision curve analyses of the clinical benefit rate.

Univariate and multivariate Cox analyses, which included the clinical factors of age, gender, type, MRI, LDH, ALB, and HGB, demonstrated that the AAG_score could serve as an independent prognostic factor for assessing MM patient outcomes (Supplementary Table 4 and Figure 6A). To develop a clinically relevant quantitative method for predicting the mortality rate of MM patients, we constructed a nomogram that incorporated the AAG_score and independent clinical prognostic factors (Figure 6B). The calibration curves for 1-, 3-, and 5-year OS for the derived nomogram and the ideal model demonstrated that the predictions were highly accurate (Figure 6C). Furthermore, the DCA curves showing the clinical utility of each model indicated that the nomogram had the greatest net benefit for survival prediction, followed by the AAG_score, while other variables were less effective (Figure 6D–6F).

### Associations of the AAG_score with the immune microenvironment and the immunotherapeutic response

**Figure 7:**
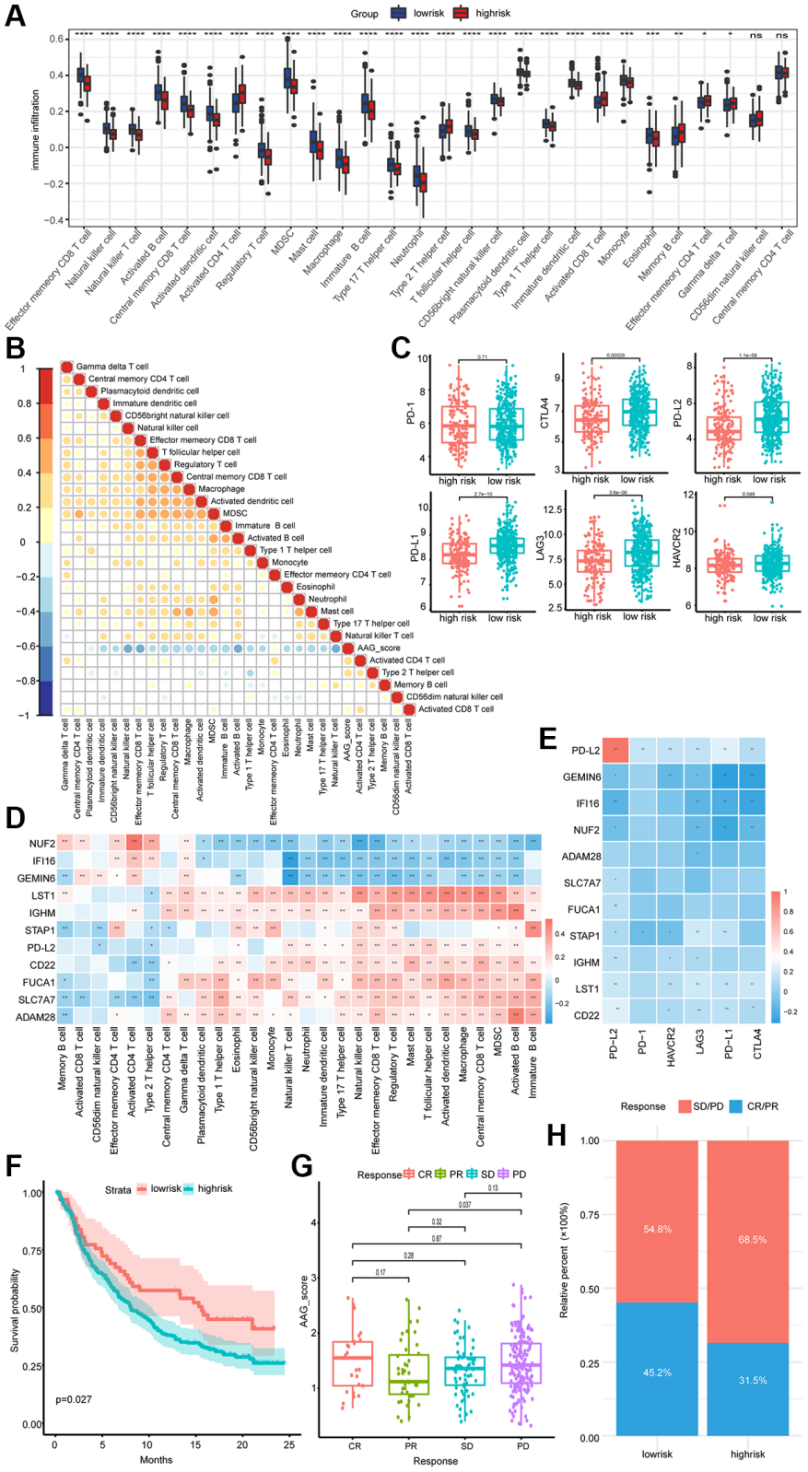
**Association of the AAG_score with the immune microenvironment and immunotherapeutic response.** (**A**) Differences in the levels of 28 infiltrating immune cells between the low-risk and high-risk groups (^*^P < 0.05; ^**^P < 0.01; ^***^P < 0.001; ^****^P < 0.0001). (**B**) The correlation between the AAG_score and the infiltration of 28 immune cell types. Blue, negative correlation; orange, positive correlation. (**C**) Expression of 6 ICPs in the low-risk and high-risk groups. (**D**) Correlations between the 11 selected molecules and 28 infiltrating immune cell types. Red, positive; blue, negative. (**E**) The correlation between the 11 selected molecules and 6 ICPs. (**F**) OS analyses of the high- and low-AAG_score groups in the anti-PD-L1 immunotherapy cohort using Kaplan–Meier curves. (**G**) Differences in the AAG scores of patients in different anti-PD-L1 clinical response groups. (**H**) The proportion of patients in the high- and low-AAG_score groups who responded to anti-PD-L1 immunotherapy.

We then investigated the correlation between 28 immune metagenes and the AAG_score. Activated CD4+ T cells, activated CD8+ T cells, memory B cells, effector memory CD4+ T cells, and Gramma delta T cells exhibited markedly increased infiltration in the high-risk groups; CD56 dim natural killer cells and central memory CD4+ T cells showed no significant difference between the two groups, while the other cell types presented the opposite trend (Figure 7A). We also analyzed the correlation between the AAG_score and each immune cell type. In addition to the correlation between the AAG_score and effector memory CD4 T cells, there was a significant negative correlation between the AAG_score and immune cell abundance (Figure 7B). The expression status of 6 ICPs was explored, and the majority of the patients in the low-risk group were highly expressed (Figure 7C).

In addition, considering the significant role of the AAG_score in immune cell infiltration, we correlated 11 molecules with the abundance of immune infiltrating cells. Spearman correlation analyses revealed that LST1, IGHM, PD-L2, CD22, FUCA1, SLC7A7, and ADAM28 exhibited prominent positive correlations with most of the immune cell infiltrates, while NUF2, IFI16, and GEMIN6 presented the opposite trend (Figure 7D). We also found that the expression of 11 key molecules was negatively correlated with that of almost all immune checkpoint molecules, and GEMIN6, LST1, STAP1, and CD22 showed particularly significant correlations (Figure 7E).

Immunotherapy is considered the standard of care for the treatment of MM. Using data from the IMvigor210 cohort receiving anti-PD-L1 immunotherapy, we investigated the ability of the AAG_score to predict the clinical response of patients to immune checkpoint blockade therapy. As depicted in Figure 7F, patients in the low-risk subgroup had a better prognosis than patients in the high-risk subgroup did. Patients with progressive disease exhibited a greater risk than did those with a partial response (Figure 7G). We also observed an enhanced therapeutic response to PD-L1 blockade therapy in low-risk patients compared to high-risk patients, implying that the low-risk group may be more sensitive to immunotherapy (Figure 7H).

## DISCUSSION

Angiogenesis is a characteristic of MM progression through the transition from monoclonal gammopathies of undetermined significance (MGUS) to MM and involves either direct production of angiogenic molecules by plasma cells or their induction within the BM microenvironment [31, 32]. Angiogenic cytokines have prognostic potential and are important factors in regulating immunity. These factors can directly promote the enrichment of MDSCs, tumor-associated macrophages (TAMs), and regulatory T cells (Tregs) and mediate immune suppression. All of these immune populations, in turn, promote angiogenesis [33]. Although MM is still incurable, treatment breakthroughs may be achieved due to the emergence of many new immunotherapies [34]. The combination of antiangiogenic therapies with immune checkpoint inhibitors has become an attractive strategy, although there is still a long way to go, considering immune escape, toxicity, side effects, etc., [35, 36]. Therefore, joint analysis of angiogenic factors and immune molecular subtypes is highly clinically important.

In this study, we determined the transcriptional changes and mutation intensities of AAGs according to the GEO and MMRF cohorts. Although the frequency of AAG mutations was low, most AAG hub genes were upregulated in MM patients. Immunohistochemistry and K–M analyses confirmed that a high MVD was associated with unfavorable clinical outcomes. The correlation between prognosis and VEGFA, which was predicted to be one of the most significantly upregulated hub genes in MM, was consistent with the correlation between prognosis and MVD. We subsequently divided the MM patients into three angiogenesis subgroups based on 35 AAGs, and these subgroups exhibited significant differences in clinical outcome, biological functions, immune infiltration, and immune checkpoints. These results suggest that AAGs are critical for screening immune molecular subtypes and evaluating the response to checkpoint immunotherapy. Therefore, we established an AAG_score model with 11 selected immunity- and prognosis-associated DEGs to quantify angiogenesis subgroups using LASSO Cox regression analysis. There is growing evidence that a single key molecule can induce immune tolerance by altering the infiltration of immune cells in the tumor microenvironment and mediating escape from immune surveillance by remodeling the tumor structure [37–39]. We thus explored whether these 11 key molecules regulate the infiltration of immune cells. We found a significant positive correlation between favorable prognosis-related molecules (LST1, IGHM, PD-L2, CD22, FUCA1, SLC7A7, and ADAM28) and the infiltration of most immune cells, while the relationships between risk-related molecules (NUF2, IFI16, and GEMIN6) and immune cell infiltration were the opposite. We also revealed that the expression of 11 molecules was negatively correlated with that of almost all the ICPs, indicating that these key molecules could mediate the occurrence of individual infiltration patterns of immune cells as well as the response to immune checkpoint blockade.

To verify the independence and clinical utility of the AAG_score, multiple analyses were performed. The AAG_scores among the different AAG_clusters and gene clusters were significantly different; AAG_cluster C and gene cluster B, which had the best clinical outcomes, had the lowest AAG_scores, revealing that the AAG_score was positively correlated with unfavorable prognosis. ROC curves validated the robustness of the model for predicting 1-, 3-, and 5-year OS. Univariate and multivariate Cox analyses indicated that the AAG_score was an independent prognostic biomarker in MM patients. The nomogram based on the AAG_score and other independent prognostic factors performed well compared to the ideal model, and its net benefit for survival prediction was greater than that of other factors.

The immune microenvironment and aberrant angiogenesis in MM form a permissive ecosystem that supports disease progression via angiogenic factors released through multiple pathways [40–43]. Thus, we evaluated the overall immune profiles of patients in different molecular clusters. AAG_cluster C was linked to a highly activated immune status in the BM microenvironment and exhibited the best prognosis among the three AAG_clusters. Similarly, the low-risk group exhibited an enhanced immune status. These findings suggested that the immune-enriched subtypes may correspond to a better prognosis in MM patients, which is concordant with the findings of a previous report [44]. The development of immune checkpoint inhibitors has provided new options for immunotherapy in MM, but the limited response to monotherapy and the accompanying adverse drug reactions have become the main obstacles to clinical application [45]. This may be because the levels of ICPs expression in high-risk and relapsed MM cells are relatively low [46, 47]. It is possible to achieve the effects of reducing side effects and improving the efficacy of drugs by altering the expression of ICPs [48], combining them with other drugs [49, 50], or providing precise treatment for the individual differences in the expression of ICPs. Hence, we further explored the correlation between different subgroups and 6 immune checkpoint molecules (PD-1, PD-L1, PD-L2, CTLA4, TIM-3, and LAG3), which are highly relevant therapeutic targets. Our results indicated that the expression levels of PD-1, PD-L2, CTLA4, TIM-3, and LAG3 in the low-risk group were significantly greater than those in the high-risk group. Consistent with the findings of the ICP cohort, patients in the IMvigor210 cohort with a low AAG_score exhibited a markedly enhanced clinical response to anti-PD-L1 immunotherapy and presented a significant survival benefit. Therefore, we concluded that angiogenesis has a strong influence on the immune phenotype and that the AAG_score has the potential to help clinicians choose precise immune checkpoint blockade treatments, which may contribute to the realization of personalized medicine.

Undeniably, this study has several limitations. The first one is the insufficiency of elucidating the mechanism of these AAGs. How they function in shaping the immune microenvironment remains unclear and needs further study. Moreover, a larger sample size is needed to further validate this model.

## CONCLUSION

In conclusion, this study explored the correlation between angiogenesis and the clinical features, prognosis, immunophenotype, and immunotherapeutic response of MM patients. We constructed an AAG_score model to predict patient prognosis and evaluate the immune microenvironment, and this model presented great robustness and independence. Comprehensive evaluation of MM patients’ AAG_scores could guide physicians in selecting more effective immunotherapy strategies and provide a basis for individualized treatment and breakthroughs in MM treatment.

## Supplementary Materials

Supplementary Figures

Supplementary Tables 1, 3 and 4

Supplementary Table 2
