# Supplementary material for: Identification of an angiogenesis-related risk score model for survival prediction and immunosubtype screening in multiple myeloma
Source: Aging (Albany NY). 2024 Feb 5;16(3):2657–78. doi: 10.18632/aging.205502 (PMC10911366; doi:10.18632/aging.205502)
Supplement: Supplementary Figures [file aging-16-205502-s001.pdf]

SUPPLEMENTARY FIGURES

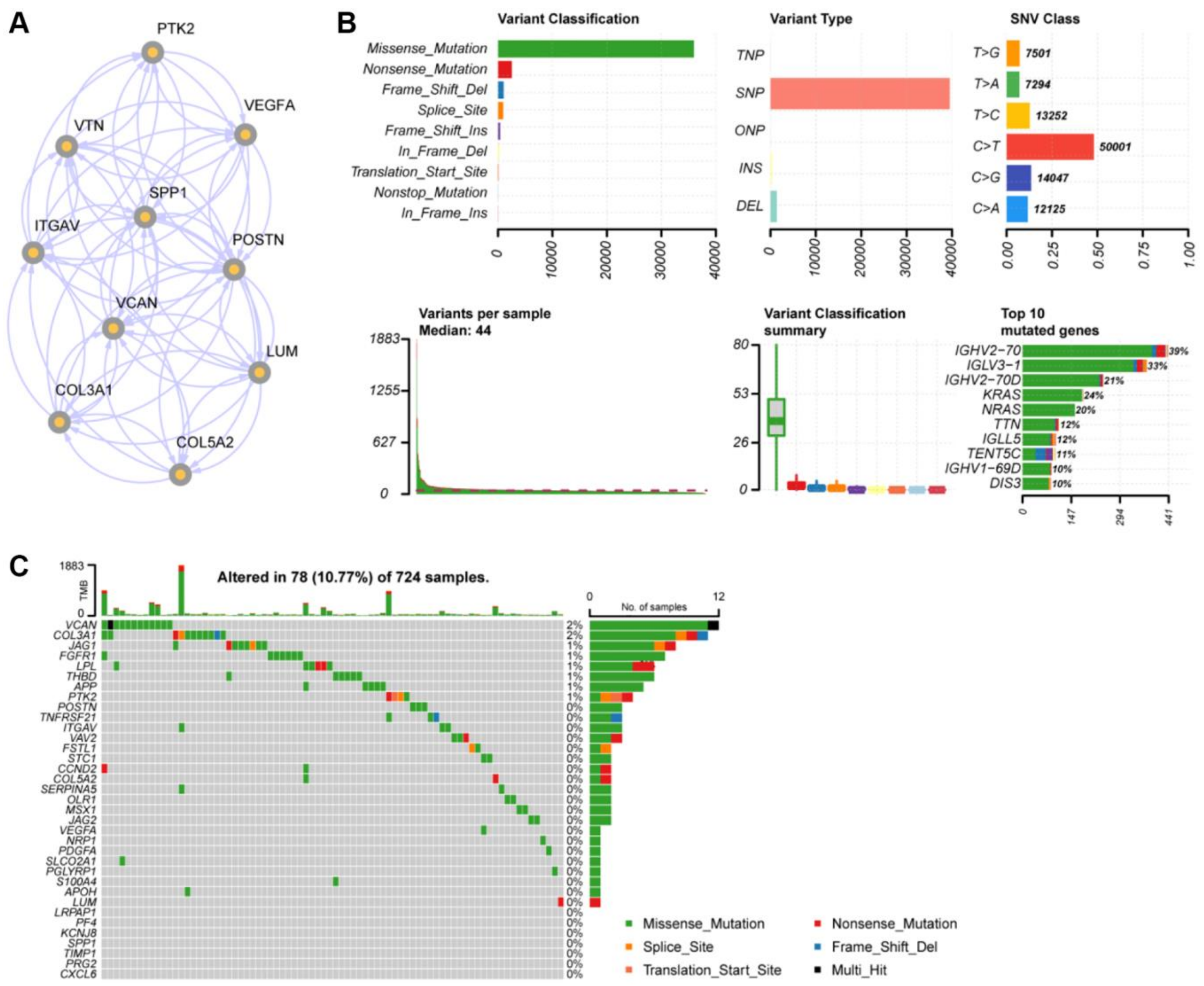

Supplementary Figure 1. PPI network and mutational landscape of AAGs. (**A**) Construction of PPI network of hub genes using the MCODE plugin in Cytoscape. (**B**) Variant classifications of mutations in MM. (**C**) Genetic alteration on a query of AAGs.

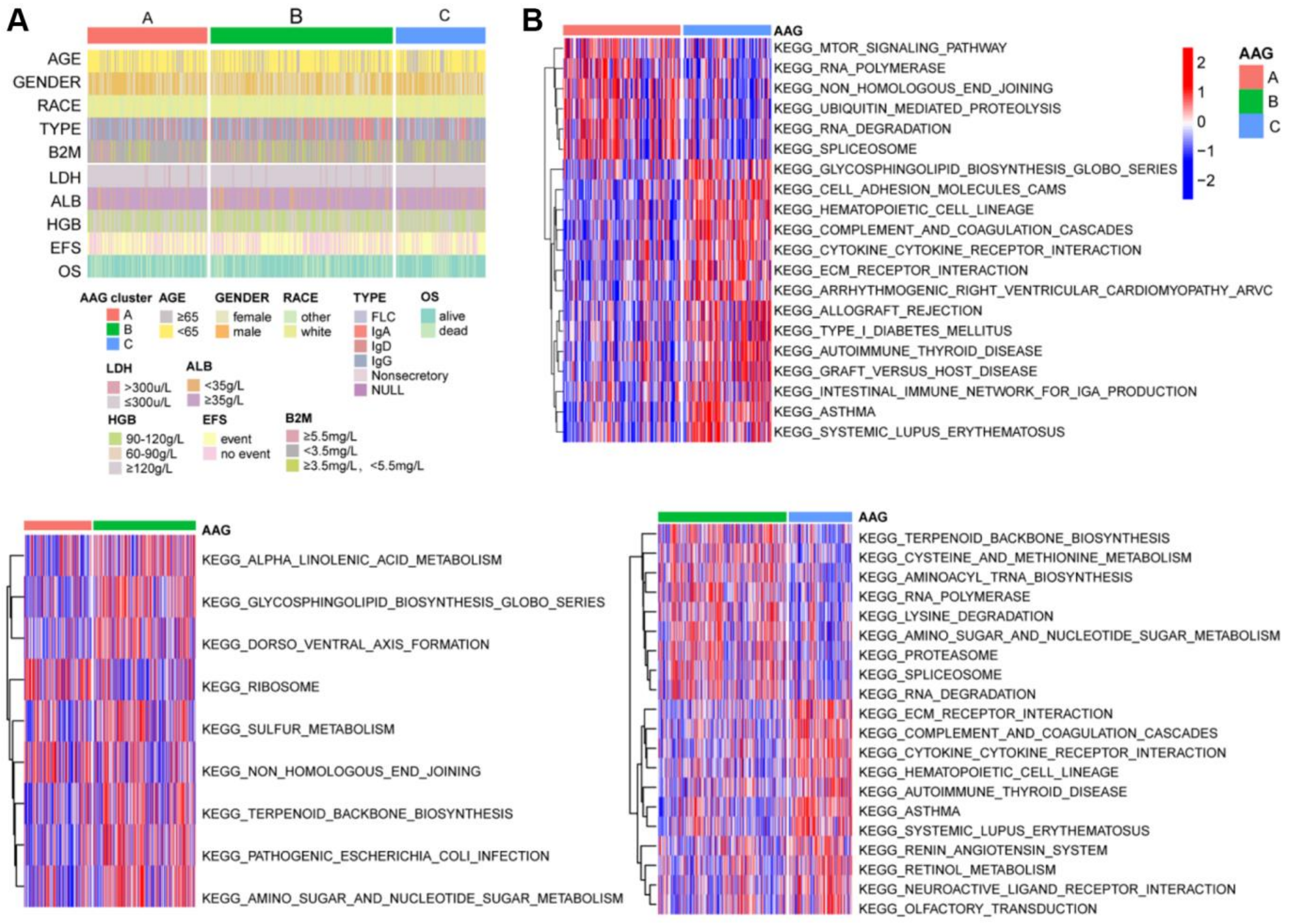

Supplementary Figure 2. Clinical characteristics and GSVA of key pathways among three AAG_clusters. (**A**) Cluster diagram of clinical characteristics among the three AAG_clusters. (**B**) GSVA of biological activities among three distinct subgroups.

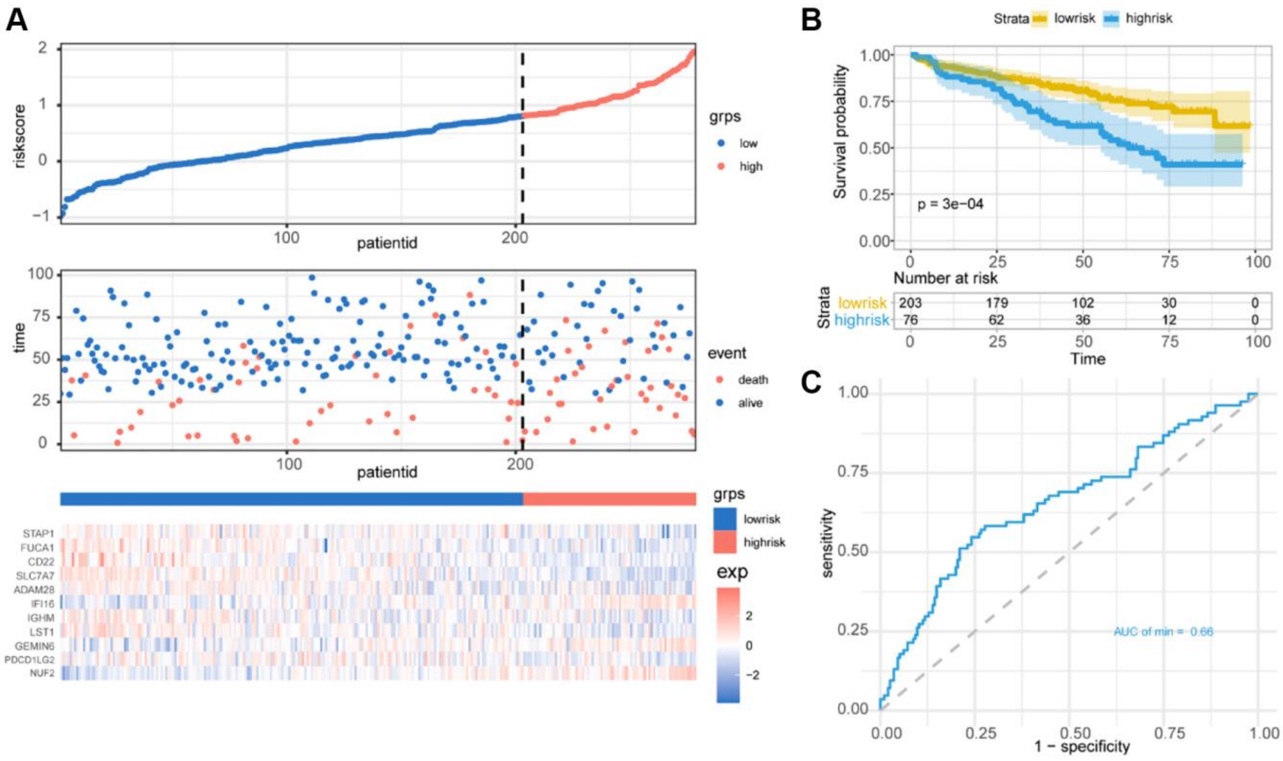

Supplementary Figure 3. Validation of AAG_score in the test cohort. (**A**) Percentage of deaths in high and low-risk groups as AAG_score values increased. Expression patterns of 11 selected molecules in different risk groups. (**B**) Overall survival analysis of risk groups using Kaplan-Meier curves. (**C**) The predictive value of AAG_score for predicting the OS of MM patients.

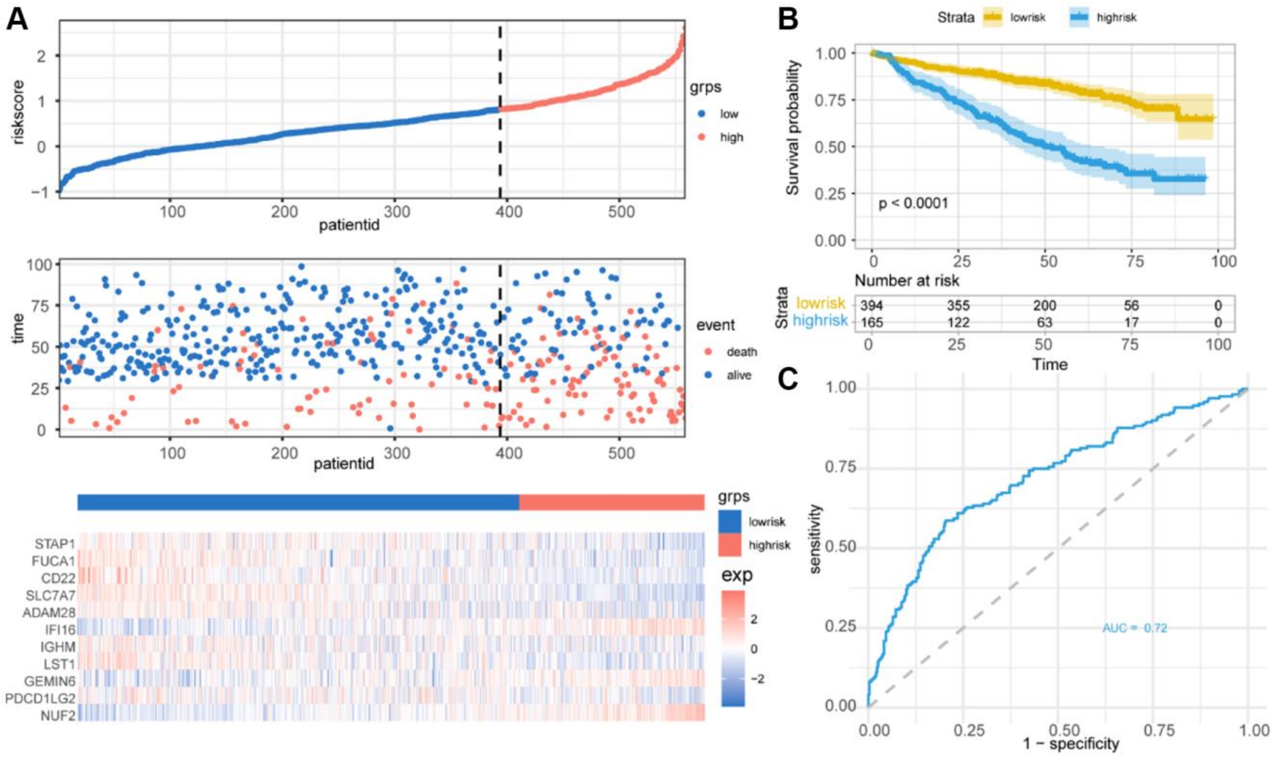

Supplementary Figure 4. Validation of AAG_score in the entire cohort. (**A**) Percentage of deaths in high and low-risk groups as AAG_score values increased. Expression patterns of 11 selected molecules in different risk groups. (**B**) Overall survival analysis of risk groups using Kaplan-Meier curves. (**C**) The predictive value of AAG_score for predicting the OS of MM patients.
