# Supplementary material for: Identification of an angiogenesis-related risk score model for survival prediction and immunosubtype screening in multiple myeloma
Source: Aging (Albany NY). 2024 Feb 5;16(3):2657–78. doi: 10.18632/aging.205502 (PMC10911366; doi:10.18632/aging.205502)
Supplement: Supplementary Tables 1, 3 and 4 [file aging-16-205502-s002.pdf]

SUPPLEMENTARY TABLES

Supplementary Table 1. Summary of 36 recognized angiogenesis-associated genes.

Gene	Type
VCAN	Angiogenesis
POSTN	Angiogenesis
FSTL1	Angiogenesis
LRPAP1	Angiogenesis
STC1	Angiogenesis
LPL	Angiogenesis
VEGFA	Angiogenesis
PF4	Angiogenesis
THBD	Angiogenesis
FGFR1	Angiogenesis
TNFRSF21	Angiogenesis
CCND2	Angiogenesis
COL5A2	Angiogenesis
ITGAV	Angiogenesis
SERPINA5	Angiogenesis
KCNJ8	Angiogenesis
APP	Angiogenesis
JAG1	Angiogenesis
COL3A1	Angiogenesis
SPP1	Angiogenesis
NRP1	Angiogenesis
OLR1	Angiogenesis
PDGFA	Angiogenesis
PTK2	Angiogenesis
SLCO2A1	Angiogenesis
PGLYRP1	Angiogenesis
VAV2	Angiogenesis
S100A4	Angiogenesis
MSX1	Angiogenesis
VTN	Angiogenesis
TIMP1	Angiogenesis
APOH	Angiogenesis
PRG2	Angiogenesis
JAG2	Angiogenesis
LUM	Angiogenesis
CXCL6	Angiogenesis

Supplementary Table 3. Prognostic analysis of 11 key molecules with a univariate Cox regression model.

Genes	HR	95% CI	*p*-value
IFI16	2.1	1.6–2.7	2.10E-08
STAP1	0.81	0.73–0.9	0.00011
GEMIN6	1.6	1.3–1.9	4.20E-05
SLC7A7	0.62	0.52–0.74	2.10E-07
LST1	0.83	0.75–0.92	0.00048
IGHM	0.92	0.87–0.97	0.0027
FUCA1	0.72	0.59–0.88	0.0017
PDCD1LG2	0.85	0.74–0.98	0.02
NUF2	1.4	1.2–1.5	7.60E-09
CD22	0.82	0.7–0.97	0.023
ADAM28	0.94	0.89–0.99	0.018

Supplementary Table 4. Prognostic analysis of clinical features with a univariate Cox regression model.

Clinical Features	Overall survival			Event-free survival		
	HR	95% CI	*p*-value	HR	95% CI	*p*-value
AGE	1.02	1.00–1.04	0.0046	1.014	1–1.027	0.05
Gender	0.96	0.71–1.31	0.81	0.9988	0.7746–1.288	0.99
B2M	1.083	1.065–1.102	9.50E-20	1.072	1.055–1.089	1.30E-17
LDH	1.006	1.005–1.008	1.60E-13	1.005	1.004–1.007	7.20E-11
ALB	0.58	0.47–0.71	1.90E-07	0.69	0.57–0.83	8.80E-05
HGB	0.87	0.8–0.94	8.70E-04	0.87	0.82–0.94	1.20E-04
MRI	1.018	1.009–1.026	8.20E-05	1.011	1.003–1.019	7.50E-03
AAG_score	2.883	2.256–3.685	2.00E-16	2.536	2.06–3.121	2.00E-16
**FLC**		**Reference**			**Reference**	
IgA	1.075	0.6545–1.767	7.74E-01	1.413	0.92447–2.160	1.10E-01
IgD	8.54E-01	0.1155–6.323	0.878	3.507	1.06999–11.496	3.83E-02
IgG	1.036	0.6634–1.619	8.75E-01	1.187	0.80446–1.752	3.88E-01
Nonsecretory	1.12E-07	NULL	9.93E-01	2.82E-01	0.03846–2.065	2.13E-01
